# Pregnancy Outcomes after Treatment for Cervical Cancer Precursor Lesions: An Observational Study

**DOI:** 10.1371/journal.pone.0165276

**Published:** 2017-01-04

**Authors:** Sheila Weinmann, Allison Naleway, Geeta Swamy, Girishanthy Krishnarajah, Bhakti Arondekar, Jovelle Fernandez, Evan Myers

**Affiliations:** 1 The Center for Health Research, Kaiser Permanente Northwest, Portland, Oregon, United States of America; 2 Department of Obstetrics & Gynecology, Duke University Medical Center, Durham, North Carolina, United States of America; 3 US Health Outcomes and Medical Policy, GlaxoSmithKline, Philadelphia, Pennsylvania, United States of America; 4 North American Vaccine Development, GlaxoSmithKline Biologicals, Philadelphia and King of Prussia, Pennsylvania, United States of America; Fondazione IRCCS Istituto Nazionale dei Tumori, ITALY

## Abstract

**Objective:**

To examine whether surgical procedures involving the uterine cervix were associated with pregnancy outcomes, including preterm birth, low birth weight, cesarean delivery and pregnancy loss.

**Design:**

Population-based observational study nested in retrospective matched cohort

**Setting:**

Kaiser Permanente Northwest integrated health plan in Oregon/Washington, U.S.A.

**Population:**

Female health plan members age 14–53 years with documented pregnancies from 1998–2009. Women with prior excisional and ablative cervical surgical procedures (N = 322) were compared to women unexposed to cervical procedures (N = 4,307) and, separately, to those having undergone only diagnostic/biopsy procedures (N = 847).

**Methods:**

Using log-linear regression models, we examined risk of adverse pregnancy outcomes in relation to prior excisional or ablative cervical surgical procedures. We stratified excisional procedures by excision thickness. We evaluated for confounding by age, body mass index, race, smoking history, previous preterm birth, and parity.

**Results:**

We found a positive association between excisional treatment > = 1.0 cm and the outcomes preterm birth and low birth weight (preterm birth unadjusted risk ratio [RR] = 2.15, 95% confidence interval [CI] 1.16–3.98 for excisions ≥1.0 cm compared to unexposed women), particularly in women who delivered within one year of surgery (RR = 3.26, 95% CI 1.41–7.53). There was no clear association between excisional treatment and cesarean delivery, and treated women did not have a substantially higher risk of dysfunctional labor. Ablative treatment was not associated with low birth weight, preterm birth, or cesarean delivery but was associated with pregnancy loss (RR = 1.43, 95% CI 1.05–1.93 vs. unexposed women). Analyses using the diagnostic procedures comparison group produced similar results.

**Conclusion:**

Women with > = 1.0 cm excisional treatment had elevated risk of preterm birth and low birth weight when compared to unexposed women and women with cervical diagnostic procedures. This suggests that increased risk derives from the treatment itself, not from other characteristics. The observed association between pregnancy loss and ablative surgical treatment requires further investigation.

## Introduction

When high-grade cervical intraepithelial neoplasia (CIN) is detected through Papanicolaou (Pap) screening and follow-up colposcopy or colposcopy-directed diagnostic biopsy, gynecologists may proceed to remove abnormal tissue before it progresses to cervical cancer [1]. Surgical procedures for CIN lesion removal are categorized as excisional or ablative. Cervical surgeries are not uncommon and are usually performed during women’s reproductive years. Adverse pregnancy outcomes, including preterm birth, low birth weight, premature rupture of membranes, cesarean delivery, and pregnancy loss, have been linked to cervical surgical treatment in some studies [2–9], but others contend that these associations may be due rather to risk factors associated with CIN [10].

The mechanisms by which cervical treatments may adversely affect pregnancy outcomes include resection of a large volume of cervical stroma, which could compromise the cervix’s structural integrity [11]; pathophysiologic changes leading to breakdown of membrane collagen; or immunologic factors, such as defense mechanism impairment or distortion of cervicovaginal flora [12–14].

Our study objective was to examine whether women with excisional and ablative surgical procedures involving the uterine cervix experienced preterm birth, low birth weight, cesarean delivery, and pregnancy loss compared to women with and without CIN in a large defined health plan population.

## Materials and Methods

### Study design

We constructed a retrospective matched cohort of women age 14–53 years during the period 1998–2009 who were members of Kaiser Permanente Northwest (KPNW). This analysis included cohort women with documented live birth and examined differences in pregnancy outcomes between women who had previously undergone cervical surgical procedures and women who were not exposed to cervical surgical or diagnostic procedures and, as a separate comparison group, women who were exposed only to cervical diagnostic/biopsy procedures. We also examined, in women with documented pregnancy, whether cervical surgical procedures were associated with risk of pregnancy loss.

KPNW is an integrated health plan serving northwest Oregon and southwest Washington with a similar demographic profile to the surrounding community. The KPNW Institutional Review Board approved the study protocol with waiver of informed consent.

### Subjects

Using the KPNW membership database, we identified female health plan members who were age 14–53 years during the years 1998–2009. Details of cohort construction were described previously [15]. We classified women according to whether their electronic medical records (EMR) showed evidence of cervical surgical treatment or a cervical diagnostic procedure (colposcopy and/or biopsy), identified by a list of International Classification of Disease 9^th^ Revision (ICD-9) and Current Procedural Terminology (CPT) codes. We used ICD-9 and CPT codes to classify surgical treatments as either excisional or ablative (S1 Table). Excisional procedures included loop electrosurgical excision procedure (LEEP or LLETZ) and conization (cold knife, laser, or loop electrode). Ablative procedures included laser ablation, cryosurgery, and cautery (electro or thermal). All women were required to have at least six continuous months of health plan membership before the treatment or diagnostic procedure.

We selected women with diagnostic procedures as a comparison group, because they, like the treatment group, were likely to have been infected with human papillomavirus (HPV) with resulting abnormal Pap tests, and they may be similar to the treatment group in other unmeasured factors.

To construct a second comparison group, we randomly assigned a study index year to each woman who met the study inclusion criteria and had not been exposed to any cervical diagnostic or treatment procedure during the study period. We frequency-matched unexposed women to treated women by index year and five-year age category. Within each year-age stratum, we randomly assigned index dates of treated women to unexposed women. We excluded unexposed women who were not enrolled in the health plan on their assigned index date and for at least six continuous months previously. In all three groups, we excluded women with hysterectomy, oophorectomy, sterilization, and genetic infertility diagnosis as of their index date (date of procedure or assigned index date for unexposed). Our final matching ratio of treated to unexposed women was 1:20.

After selecting the treatment, diagnostic-only, and unexposed cohorts, we restricted the study population to women with documented live birth, medically-attended spontaneous abortion, or stillbirth, using a tested and validated set of SAS programs and pregnancy indicator codes developed for use in KPNW datasets [16]. Women who left the health plan before these events were not included in analysis. For the live birth analyses, the first singleton live birth during the study period was included for each woman. For the pregnancy loss analysis, we used the first pregnancy in the study period.

### Data collection

We used KPNW electronic data files to obtain health plan enrollment, diagnosis, procedure, pharmacy vital sign, and demographic data for the period 1/1/1997 through 12/31/2009. Variables selected for evaluation as confounders or effect modifiers included age, race/ethnicity, BMI, cigarette smoking status, parity, previous preterm birth, and Medicaid status. Where race/ethnicity and BMI data were missing, we imputed values for these variables. Women with missing BMI were categorized by age group and assigned the mean BMI value of that age group. Most missing values for race/ethnicity were replaced by geo-coded race/ethnicity information from the U.S. 2000 Census. The values that were still missing after utilizing the geo-coded information were replaced by the modal value “white”.

For women with excisional treatment procedures, an experienced medical record technician determined thickness [17] of cervical excision from the pathology report. If, in addition to a regular LEEP, a woman had an endocervical (“top hat”) LEEP procedure, we summed the two thickness measurements according to the method described by Ortoft *et al* [8].

Our pregnancy outcomes of interest included medically-attended spontaneous abortion before 20 weeks gestation, stillbirth, gestational age, birth weight, and cesarean delivery. To determine all outcome variables, we used the KPNW pregnancy algorithm, which searches for potential pregnancy indicators using ICD-9 and CPT codes as well as laboratory tests, pharmacy dispensings, and imaging procedures associated with pregnancy; in previous work, this algorithm has been validated against data manually abstracted from medical records by trained research staff [15]. To reduce the amount of missing birth weight data, we collected birth weight from the State of Oregon birth certificate files. Where these data were still missing, an experienced medical record technician reviewed the KPNW medical record to locate this information. If birth weight seemed implausible for gestational age, we reviewed the medical records. If birth weight was still undetermined, we excluded the mothers from the low birth weight, preterm birth, and cesarean delivery analyses so that all live birth analyses would be conducted on the same population. We collected information on reasons for cesarean delivery by searching for relevant CPT codes within 7 days post-delivery.

### Data management and statistical methods

We used SAS statistical software Version 9.2 (SAS Institute, Cary, NC) to select cohort members and manage and analyze data. Study team members reviewed datasets for inconsistencies and missing information and reviewed descriptive statistics to detect anomalies or temporal changes.

For all cohort women, we developed propensity scores describing likelihood of exposure to cervical surgical treatment. Using logistic regression analysis, we computed separate propensity score models for treatment vs. unexposed and treatment vs. diagnosis-only. All variables in the propensity scores were measured for the time period before the index date.

We examined preterm birth, low birth weight, and cesarean delivery as dichotomous (yes/no) variables. Using standard medical definitions, preterm birth was defined as delivery prior to 37 weeks gestation, and low birth weight was defined as birth weight below 2,500 grams. For the pregnancy loss analysis, women with either spontaneous abortion or stillbirth were classified as having pregnancy loss.

We carried out descriptive analyses and calculated comparison statistics to separately compare women in the treatment group with the unexposed group and the diagnostic-only group. In both comparisons, we removed subjects in each exposure group whose propensity scores did not overlap with scores of comparison group subjects [18]. Women with surgical treatment were evaluated all together and also stratified according to type of treatment (excisional versus ablative). Women with excisional procedures were further stratified according to excision thickness (<1.0 cm versus ≥1.0 cm) using a three-level predictor variable. We also divided excision thickness into four groups (0.1–0.5, 0.6–1.0, 1.1–1.5, 1.6+ cm) for descriptive analysis.

We calculated prevalence ratios or risk ratios (RRs) using log-linear regression models. When log-linear models did not converge, we used Poisson models. We calculated univariate models and assessed for confounding by determining whether potential confounders including age group, BMI category, race, smoking history, previous preterm pregnancy, and parity altered the risk ratio by ≥10%.

## Results

From a population of 461,084 women, 100,577 (4,138 treated, 82,760 unexposed, and 13,679 diagnostic-only) met study eligibility and matching criteria, constituting the matched cohort. After selecting the 5,888 cohort members who delivered a singleton live birth during the study period, we removed 345 women (6 treated, 338 unexposed, and 1 diagnostic-only) from the analysis because their propensity scores did not overlap with the propensity score distribution of the comparison group, suggesting very different patterns of risk factors. Sixty-seven women were excluded due to missing infant birth weight. The final analytic groups included 322 women with surgical treatment (229 excisional and 93 ablative), 847 women with diagnostic procedures only, and 4,307 women unexposed to either procedure during the study period. Among 229 women with excisional treatment, we had excision thickness data for 199 (87%). Of all pathology reports reviewed for excision thickness, 69% had excisions < 1.0 cm, 8% had 1.0 cm excisions, and 23% had excisions >1.0 cm.

Among women with live births, women who underwent surgical treatment were more likely than unexposed women to smoke (34% vs. 21%), be nulliparous (44% vs. 31%), and have BMI <25 (53% vs. 40%). Women with only diagnostic procedures were similar to treated women on these factors (Table 1).

**Table 1:** Demographic and behavioral characteristics of women who had undergone previous cervical surgical treatment compared with those who were either unexposed to cervical procedures or had only undergone diagnostic treatment, Kaiser Permanente Northwest, 1998–2009

Characteristic	Treatment group (n = 322)	Unexposed group (n = 4307)	Diagnostic group (n = 847)	P-value (Χ2) < .05
	n (%)	n (%)	n (%)	
**Exposure status**				
Excisional procedure	229 (71.1)	0	0	
Ablative procedure	93 (28.9)	0	0	
**Age in yrs (mean ± SD)**	26.3 (5.1)	26.8 (5.1)	25.6 (6.0)	
**Age**				*,+
< 20 yrs	38 (11.8)	304 (7.1)	163 (19.2)	
20–24 yrs	81 (25.2)	1080 (25.1)	218 (25.7)	
25–29 yrs	116 (36.0)	1684 (39.1)	242 (28.6)	
30–34 yrs	70 (21.7)	967 (22.5)	153 (18.1)	
35+ yrs	17 (5.3)	272 (6.3)	71 (8.4)	
**White race**				*,+
Yes	288 (89.4)	3625 (84.2)	701 (82.8)	
No	30 (9.3)	576 (13.4)	129 (15.2)	
Unknown	4 (1.2)	106 (2.5)	17 (2.0)	
**Medicaid recipient**				
Yes	18 (5.6)	185 (4.3)	57 (6.7)	
No	304 (94.4)	4122 (95.7)	790 (93.3)	
**Smoking status**				*
Ever Smoker	110 (34.2)	885 (20.6)	300 (35.4)	
Never Smoker	212 (65.8)	3422 (79.5)	547 (64.6)	
**BMI**				*
Underweight (< 18.5)	8 (2.5)	68 (1.6)	16 (1.9)	
Normal (18.5–24.9)	162 (50.3)	1651 (38.3)	430 (50.8)	
Overweight (25.0–29.9)	85 (26.4)	1682 (39.1)	244 (28.8)	
Obese (30.0–39.9)	60 (18.6)	746 (17.3)	123 (14.5)	
Extremely obese (> = 40)	7 (2.2)	160 (3.7)	34 (4.0)	
**Previous preterm birth**				
Yes	28 (8.7)	400 (9.3)	89 (10.5)	
No	294 (91.3)	3907 (90.7)	758 (89.5)	
**Previous births**				*
1 or more	156 (48.5)	2703 (62.8)	437 (51.6)	
None	142 (44.1)	1335 (31.0)	362 (42.7)	
Unknown	24 (7.5)	269 (6.3)	48 (5.7)	

* Surgical treatment group compared with unexposed group.

+ Surgical treatment group compared with diagnostic procedure only group.

Note: Race imputed for 17%, 42%, and 25% of treated, unexposed, and diagnostic-only groups respectively, using geocoding based on census tract of residence. Body mass index imputed for 11%, 38%, and 9% of treated, unexposed, and diagnostic-only groups respectively, based on the mean BMI values in the age groups above.

We observed no significant confounding by age, BMI, race, smoking history, previous preterm birth, or parity. For completeness, both univariate and multivariable models are presented in Tables 2 and 3. Because of small numbers, we did not compute multivariable models for the analyses stratified on excision thickness.

**Table 2:** Risk of preterm birth, low birth weight, and cesarean delivery in 322 women with cervical surgical treatment compared to 4,307 women unexposed to cervical treatment or diagnostic procedures, Kaiser Permanente Northwest, 1998–2009.

TREATMENT TYPE	PRETERM BIRTH			LOW BIRTH WEIGHT			CESAREAN DELIVERY		
	Treatment group	Unexposed group	RR (95% CI)	Treatment group	Unexposed group	RR (95% CI)	Treatment group	Unexposed group	RR (95% CI)
	N (%)	N (%)		N (%)	N (%)		N (%)	N (%)	
**Any treatment**									
Unadjusted1	27 (8.4)	286 (6.6)	1.26 (0.87–1.84)	21 (6.5)	178 (4.1)	1.58 (1.02–2.44)	97 (30.1)	1186 (27.5)	1.09 (0.92–1.30)
Unadjusted2	25 (8.5)	258 (6.5)	1.30 (0.88–1.93)	19 (6.5)	160 (4.0)	1.60 (1.01–2.53)	87 (29.6)	1088 (27.4)	1.08 (0.90–1.29)
Adjusted	25 (8.5)	258 (6.5)	1.22 (0.80–1.85)*	19 (6.5)	160 (4.0)	1.42 (0.88–2.3)*	87 (29.6)	1088 (27.4)	1.04 (0.87–1.24)
**Ablative treatment**									
Unadjusted1	5 (5.4)	286 (6.6)	0.81 (0.34–1.91)	6 (6.5)	178 (4.1)	1.56 (0.71–3.43)	20 (21.5)	1186 (27.5)	0.78 (0.53–1.16)
Unadjusted2	5 (6.0)	258 (6.5)	0.91 (0.39–2.15)	6 (7.1)	160 (4.0)	1.76 (0.8–3.87)	17 (20.2)	1088 (27.4)	0.74 (0.48–1.13)
Adjusted	5 (6.0)	258 (6.5)	0.70 (0.29–1.70)*	6 (7.1)	160 (4.0)	1.48 (0.65–3.38)*	17 (20.2)	1088 (27.4)	0.74 (0.49–1.12)
**Excisional treatment**									
Unadjusted1	22 (9.6)	286 (6.6)	1.45 (0.96–2.19)	15 (6.6)	178 (4.1)	1.58 (0.95–2.64)	77 (33.6)	1186 (27.5)	1.22 (1.01–1.47)
Unadjusted2	20 (9.5)	258 (6.5)	1.46 (0.95–2.25)	13 (6.2)	160 (4.0)	1.53 (0.88–2.65)	70 (33.3)	1088 (27.4)	1.21 (0.99–1.48)
Adjusted	20 (9.5)	258 (6.5)	1.49 (0.94–2.36)*	13 (6.2)	160 (4.0)	1.39 (0.79–2.47)*	70 (33.3)	1088 (27.4)	1.16 (0.96–1.41)
**Excisional <1.0 cm**									
Unadjusted	12 (8.8)	286 (6.6)	1.33 (0.77–2.31)	8 (5.9)	178 (4.1)	1.42 (0.72–2.80)	48 (35.3)	1186 (27.5)	1.28 (1.02–1.62)
**Excisional > = 1.0 cm**									
Unadjusted	9 (14.3)	286 (6.6)	2.15 (1.16–3.98)	6 (9.5)	178 (4.1)	2.30 (1.06–5.00)	22 (24.9)	1186 (27.5)	1.27 (0.90–1.78)

Unadjusted1 = all subjects without missing data for outcome variable

Unadjusted2 = all subjects without missing data for outcome variable or covariates

Adjusted = adjusted for covariates age (<20, 20–24, 25–29, 30–34, 35+), BMI (<18.5, 18.5–24.9, 25.0–29.9, 30.0–39.9, 40+), race (white/nonwhite), smoking history (ever-never), previous preterm birth (yes/no), parity (0 vs. > = 1)

Ablative and excisional RRs are unadjusted

* Log-linear regression models did not converge. Used poisson models instead.

**Table 3:** Risk of preterm birth, low birth weight, and cesarean delivery in 322 women with cervical surgical treatment compared to 847 women with cervical diagnostic/biopsy procedures only, Kaiser Permanente Northwest, 1998–2009.

TREATMENT TYPE	PRETERM BIRTH			LOW BIRTH WEIGHT			CESAREAN DELIVERY		
	Treatment group	Diagnostic group	RR (95% CI)	Treatment group	Diagnostic group	RR (95% CI)	Treatment group	Diagnostic group	RR (95% CI)
	N (%)	N (%)		N (%)	N (%)		N (%)	N (%)	
**Any treatment**									
Unadjusted1	27 (8.4)	64 (7.6)	1.11 (0.72–1.71)	21 (6.5)	35 (4.1)	1.58 (0.93–2.67)	97 (30.1)	226 (26.7)	1.13 (0.92–1.38)
Unadjusted2	25 (8.5)	59 (7.5)	1.13 (0.72–1.77)	19 (6.5)	31 (4.0)	1.63 (0.94–2.84)	87 (29.6)	211 (26.8)	1.10 (0.89–1.36)
Adjusted	25 (8.5)	59 (7.5)	1.15 (0.72–1.85)*	19 (6.5)	31 (4.0)	1.71 (0.96–3.05)*	87 (29.6)	211 (26.8)	1.11 (0.91–1.36)
**Ablative treatment**									
Unadjusted1	5 (5.4)	64 (7.6)	0.71 (0.29–1.72)	6 (6.5)	35 (4.1)	1.56 (0.67–3.61)	20 (21.5)	226 (26.7)	0.81 (0.54–1.21)
Unadjusted2	5 (6)	59 (7.5)	0.79 (0.33–1.91)	6 (7.1)	31 (4.0)	1.80 (0.78–4.2)	17 (20.2)	211 (26.8)	0.75 (0.40–1.17)
Adjusted	5 (6)	59 (7.5)	0.79 (0.31–1.98)*	6 (7.1)	31 (4.0)	1.90 (0.78–4.61) *	17 (20.2)	211 (26.8)	0.82 (0.53–1.26)
**Excisional treatment**									
Unadjusted1	22 (9.6)	64 (7.6)	1.27 (0.80–2.02)	15 (6.6)	35 (4.1)	1.59 (0.88–2.85)	77 (33.6)	226 (26.7)	1.26 (1.02–1.56)
Unadjusted2	20 (9.5)	59 (7.5)	1.26 (0.78–2.05)	13 (6.2)	31 (4.0)	1.56 (0.83–2.93)	70 (33.3)	211 (26.8)	1.24 (0.99–1.55)
Adjusted	20 (9.5)	59 (7.5)	1.30 (0.78–2.18)*	13 (6.2)	31 (4.0)	1.63 (0.85–3.15)*	70 (33.3)	211 (26.8)	1.21 (0.98–1.51)
**Excisional <1.0 cm**									
Unadjusted	12 (8.8)	64 (7.6)	1.17 (0.65–2.11)	8 (5.9)	35 (4.1)	1.42 (0.68–3.00)	48 (35.3)	226 (26.7)	1.32 (1.03–1.7)
**Excisional > = 1.0 cm**									
Unadjusted	9 (14.3)	64 (7.6)	1.89 (0.99–3.62)	6 (9.5)	35 (4.1)	2.30 (1.01–5.27)	22 (24.92)	226 (26.7)	1.31 (0.92–1.87)

Unadjusted1 = all subjects without missing data for outcome variable

Unadjusted2 = all subjects without missing data for outcome variable or covariates

Adjusted = adjusted for covariates age (<20, 20–24, 25–29, 30–34, 35+), BMI (<18.5, 18.5–24.9, 25.0–29.9, 30.0–39.9, 40+), race (white/nonwhite), smoking history (ever-never), previous preterm birth (yes/no), parity (0 vs. > = 1)

Ablative and excisional RRs are unadjusted

* Log-linear models did not converge. Used poisson models

Of the 322 treated women, 163 (51%) had the live birth within one year of treatment. In an exploratory analysis, we stratified on birth within one year after treatment (yes/no) to examine whether the effect of treatment differed by its proximity to the incident gestation.

### Preterm birth

We found a positive, though not statistically-significant, association between surgical treatment and preterm birth with elevated risk of about 20% (Tables 2 and 3). When the analysis was stratified on ablative vs. excisional treatment, only women with excisional treatment were affected, and the association was primarily among women with excisions ≥1.0 cm (treatment vs. unexposed RR = 2.15, 95% CI 1.16–3.98; treatment vs. diagnosis-only RR = 1.89, 95% CI 0.99–3.62 –both unadjusted); though the two point estimates are similar, only the treatment vs. unexposed comparison is statistically significant at p< = 0.05. Preterm babies were born to 5% of women with ablative treatment and 7–10% of women with excisional treatment <1.6 cm. Among the 18 women with excisions of ≥1.6 cm, the proportion was 28%. In comparison, 7% of unexposed and 8% of diagnostic-only women had preterm deliveries.

In stratified analysis, risk of preterm birth in women delivering within a year of excisional treatment was 1.83 (95% CI 0.90–3.71) vs. unexposed and 1.56 (95% CI 0.74–3.31) vs. diagnostic-only. Where excision thickness was > = 1.0 cm, unadjusted RRs for delivery within one year after treatment were 3.26, (95% CI 1.41–7.53) and 2.78 (95% CI 1.30–5.96) vs. unexposed and diagnostic-only respectively. We could not stratify our analysis on degree of prematurity due to small numbers of very premature infants in the surgically treated group (only four babies born before 33 weeks gestation).

### Low birth weight

Women with surgical treatment had approximately 50% higher likelihood of low birth weight than unexposed women (Table 2) and those with diagnostic procedures only (Table 3). RRs were similar when the analysis was stratified on type of treatment (ablative vs. excisional). Among women who had undergone excisional treatment, those with excision thickness ≥1.0 cm had the greatest likelihood of low birth weight (unadjusted RR = 2.30, 95% CI 1.06–5.00 and RR = 2.30, 95% CI 1.01–5.27 vs. unexposed and diagnostic-only respectively–[Tables 2 and 3]). In women who had received either ablative treatment or excisional treatment <1.6 cm, 6–7% delivered low birth weight babies. Among the 18 women with excisions ≥1.6 cm, the proportion was 11%. In comparison, 4% of both unexposed and diagnostic-only women delivered low birth weight babies.

Among women who delivered within one year of excisional treatment, RR for a low birth weight baby was 1.83 (95% CI 0.90–3.71) vs. unexposed and 2.63 (95% CI 1.13–6.13) vs. diagnostic-only. Where excision thickness was > = 1.0 cm, unadjusted RRs were 3.26 (95% CI 1.41–7.53) and 4.30 (95% CI 1.60–11.54) vs. unexposed and diagnostic-only respectively.

### Cesarean delivery

The association between cesarean delivery and surgical treatment was modestly elevated, though not statistically-significant in adjusted models, when compared with unexposed and diagnostic-only groups (Tables 2 and 3). Risk did not vary by thickness of excision except in women giving birth within one year of surgery, where RRs were higher for excision > = 1.0 cm (RR = 1.42, 95% CI 0.93–2.15 vs. unexposed and RR = 4.30, 95% CI 1.60–11.54 vs. diagnostic-only). The surgically treated group had a similar proportion of women with an ICD-9 code for dysfunctional labor (23%) as the unexposed group (19%, p = 0.38) and the diagnostic-only group (24%; p = 0.81).

### Pregnancy loss

Our cohort had 1,043 medically-attended spontaneous abortions and 22 stillbirths. In women with cervical surgery, 21% experienced pregnancy loss—25% in those with ablative and 19% in those with excisional treatment. Pregnancy loss proportions were 18% and 20% in the unexposed and diagnostic-only groups, respectively. For ablative treatment, RRs were 1.43 (95% CI 1.05–1.93) and 1.38 (95% CI 1.01–1.89) vs. unexposed and diagnostic-only respectively. For excisional treatment, there was no increased risk of pregnancy loss vs. either comparison group (Table 4). We could not stratify on trimester of pregnancy loss, due to imprecise gestational age surrounding early spontaneous abortion in our dataset. In women with surgical treatment, 7% of spontaneous abortions were classified as second or third trimester, compared to 4% and 6% in the unexposed and diagnostic-only groups respectively.

**Table 4:** Risk of pregnancy loss compared to live birth in 322 women with cervical surgical treatment compared to 4,307 women unexposed to cervical treatment or diagnostic procedures and 847 women exposed to diagnostic/biopsy procedures only, Kaiser Permanente Northwest, 1998–2009.

TREATMENT TYPE	TREATMENT VS. UNEXPOSED			TREATMENT VS. DIAGNOSTIC/BIOPSY PROCEDURES		
	Treatment group	Unexposed group	RR (95% CI)	Treatment group	Diagnostic group	RR (95% CI)
	N (%)	N (%)		N (%)	N (%)	
**Any treatment**						
Unadjusted	88 (21.0)	977 (17.7)	1.19 (0.98–1.44)	88 (21.0)	218 (19.6)	1.07 (0.86–1.33)
Adjusted	88 (21.0)	977 (17.7)	1.16 (0.96–1.41)	88 (21.0)	218 (19.6)	1.10 (0.89–1.38)
**Ablative treatment**						
Unadjusted	32 (25.2)	977 (17.7)	1.43 (1.05–1.93)	32 (25.2)	218 (19.6)	1.38 (1.01–1.89)
Adjusted	32 (25.2)	977 (17.7)	1.43 (1.06–1.93)	32 (25.2)	218 (19.6)	1.29 (0.93–1.77)
**Excisional treatment**						
Unadjusted	56 (19.1)	977 (17.7)	1.08 (0.85–1.38)	56 (19.1)	218 (19.6)	0.99 (0.76–1.29)
Adjusted	56 (19.1)	977 (17.7)	1.05 (0.83–1.34)	56 (19.1)	218 (19.6)	0.97 (0.75–1.27)

Unadjusted = All subjects without missing data for outcome variable. No subjects had missing data for covariates.

Adjusted = adjusted for covariates age (categorical), BMI (categorical), race (white/nonwhite), smoking history (ever-never).

## Discussion

We found a modestly elevated but generally not statistically-significant risk of preterm birth and low birth weight among women who had undergone prior surgical treatment for cervical dysplasia compared with two groups of women: those with no history of cervical diagnostic or treatment procedures or those with history of diagnostic procedures but no treatment. Risk was primarily associated with excisional treatment, particularly excisions > = 1.0 cm. and appeared higher for women who gave birth within a year of the surgery than for women whose babies were born later. The multivariable models’ confidence intervals generally crossed 1.0 except for the subgroup with excisions > = 1.0 cm. Surgical treatment was associated with pregnancy loss among women who had undergone ablative treatment, a finding that has not been previously reported, though not with excisional treatment. We found no significant confounding by other known predictors of adverse pregnancy outcomes.

This study is novel in that we report on four different pregnancy outcomes in the same population and in the same report. Strengths of our study included our large, population-based cohort with up to 12 years of follow-up, extensive EMR data, our previously-established pregnancy algorithm, and access to state birth records to augment information on birth weight. The EMR data were valuable for ascertaining clinical covariates, and we supplemented this data source with medical record and pathology report abstraction. We were able to establish two comparison groups for examination of treatment effects.

The similar RRs obtained for all outcomes using the two different comparison groups suggests that the increased risk associated with surgical treatment derives not from HPV exposure or demographic or behavioral characteristics associated with CIN (likely in both the treated and diagnostic-only groups), but rather from the treatment itself, though the RRs were slightly lower for most comparisons with the diagnostic-only group than for the unexposed group. Our results contrast with some previous studies [4;10;19], including a meta-analysis [10], that reported elevated risk for serious pregnancy outcomes among both treated and non-treated women with CIN. [4;10;19]

Published systematic reviews have produced variable results. Positive associations have been found between: LEEP and preterm birth [2;10] and low birth weight [2]; cold knife conization and LEEP with increased risk of preterm birth and low birth weight but no significantly-adverse results with laser ablation [3]; cold knife conization with increased risk of perinatal morality, preterm delivery and low birth weight, but no significantly increased risk with LEEP, laser conization, or laser ablation [4]. Several recent individual studies also reported an association between preterm birth and low birth weight with excisional surgery [6–8;19;20]. RRs varied widely among studies, with the highest (approximately three-fold) for cold knife conization and lower or marginal RRs for LEEP, laser conization, and laser ablation. Higher risk with greater thickness or volume of excision has been reported by most [3;8;11;21–28]; but not all [29] studies that examined this variable. One meta-analysis [19] reported a pooled RR of 1.47 (95% CI 1.24–1.74) for any ablative treatment in relation to preterm birth.

Several studies investigating preterm birth in relation to time between treatment and pregnancy have reported higher risk with a shorter interval [22;30;31] as did we, while others did not [6;32;33].

Our study found an approximately 20% elevated risk of cesarean delivery associated with excisional surgical treatment (34% in treatment group compared to 28% and 27% in the comparison groups); the associations were statistically significant for both comparison groups in unadjusted but not adjusted models. Overall, risk did not vary with excision thickness, and treated women did not have a substantially higher risk of dysfunctional labor than either comparison group. A recent study [34] reported similar results, with 32% of pregnancies after treatment resulting in Cesarean delivery, compared to 29% in Pap-only and biopsy-only groups; no increased risk with thicker excisions was found. Kyrgiou *et al*. [3] reported a three-fold increased risk in a pooled analysis of four studies of cold knife conization, and El-Bastawissi [5] reported a higher risk among women with conization (23.6% vs. 19.9% in the comparison group, p = 0.168). Otherwise, previous studies found no association [2;3;29;35–39] or decreased risk [40].

Our finding that ablative treatment is associated with an elevated risk of pregnancy loss is in conflict with the one other examination of this association (RR = 0.65, 95% CI 0.39–1.09) and requires confirmation in other studies [41]. Similar to our study, a recent meta-analysis of nine studies [42] reported no overall association between excisional treatment and miscarriage. This meta-analysis found a positive association with second trimester miscarriages (RR = 2.60, 95% CI 1.45–4.67); we were unable to stratify our analysis on trimester of pregnancy loss.

This study has several limitations. Although we had access to most treated women’s pathology reports, the thickness of the excisional procedure was not always recorded, and pathology reports were not available for some women treated outside the health plan. KPNW’s ICD-9 coding system could not precisely distinguish individual treatment procedures, limiting our ability to study the effect of specific surgical procedures on pregnancy outcomes. We had no information on untreated early spontaneous abortion, HPV exposure, sexual history, education, marital status, or socioeconomic status other than Medicaid eligibility. We lacked complete data for certain covariates, such as parity, smoking, race/ethnicity, and BMI. Although we report RRs for low birth weight, we realize that this outcome mixes preterm birth and fetal growth restriction, which likely have very different etiologies. This imprecision would likely bias study results toward the null.

In conclusion, in our large, population-based study, women who had undergone thick excisional cervical surgical procedures had an approximately doubled risk of preterm birth and low birth weight compared to two groups of women without such surgery, and this risk was higher if the baby was born within one year of surgery. The observed positive association between pregnancy loss and ablative surgical treatment has not been previously reported and requires further investigation. Cervical treatment’s association with adverse pregnancy outcomes when compared to both unexposed women and women with only diagnostic procedures suggests that the increased risk derives primarily from the treatment itself, not from other characteristics associated with HPV or CIN.

These findings suggest that efforts to minimize excision thickness in cervical surgeries are prudent. Larger studies of excision thickness in relation to adverse pregnancy outcomes are warranted, as are more studies with a CIN comparison group.

## Supporting Information

S1 Table Cervical Procedures Code List (DOC)
