# Correction: Pregnancy Outcomes after Treatment for Cervical Cancer Precursor Lesions: An Observational Study

**DOI:** 10.1371/journal.pone.0172417

**Published:** 2017-02-13

**Authors:** The PLOS ONE Staff

In the Funding section, the last sentence is incorrectly included in the statement. The publisher apologizes for the error. The correct funding information is as follows: GlaxoSmithKline Biologicals SA funded this study/research and was involved in all stages of study conduct, including analysis of the data. GlaxoSmithKline Biologicals SA also took in charge all costs associated with the development and publication of this manuscript (GSK study number: GHO-09-3981).
