# Supplementary material for: Pregnancy Outcomes after Treatment for Cervical Cancer Precursor Lesions: An Observational Study
Source: PLoS One. 2017 Jan 4;12(1):e0165276. doi: 10.1371/journal.pone.0165276 (PMC5214346; doi:10.1371/journal.pone.0165276)
Supplement: S1 Table — (DOC) [file pone.0165276.s001.doc]

**S1 Table: Cervical Procedures Code List**

***Treatment Procedures***:

| **Code** | **Code Type** | **Description** |
| --- | --- | --- |
| **Excisional procedures** | | |
| 57460 | cpt tx | Colposcopy of cervicx with loop electrode biopsy of cervix |
| 57461 | cpt tx | Colposcopy of cervix with loop electrode conization of cervix |
| 57520 | cpt tx | Conization of cervix +-d&c rpr knife/laser |
| 57522 | cpt tx | Conization of cervix +-d&c rpr eltrd exc |
| 67.2 | ICD9 tx | Conization of cervix |
| 67.32 | ICD9 tx | Destruction of lesion of cervix by caut:electro, leep,lletz |
| **Ablative procedures** | | |
| 57510 | cpt tx | Cauterization of cervix electro/thermal |
| 57511 | cpt tx | Cauterization of cervix cryocaut 1st/repeat |
| 57513 | cpt tx | Cauterization of cervix with laser ablation |
| 67.30 | ICD9 tx | Other excision or destruction of lesion or tissue of cervix |
| 67.33 | ICD9 tx | Destruction of lesion of cervix by cryosurgery |

***Diagnostic procedures***:

| **Code** | **Code Type** | **Description** |
| --- | --- | --- |
| 57420 | cpt dx | Colposcopy of entire vagina, with cervix if present |
| 57421 | cpt dx | Colposcopy of entire vagina with cervical biopsy |
| 57452 | cpt dx | Colposcopy of cervix including upper/adjacent vagina |
| 57454 | cpt dx | Colposcopy of cervix with cervix biopsy and endocervical curettage |
| 57455 | cpt dx | Colposcopy of cervix with cervix biopsy |
| 57456 | cpt dx | Colposcopy of cervix with endocervical curettage |
| 57500 | cpt dx | Biopsy of cervix 1/mlt or excision of lesion |
| 67.11 | cpt dx | Endocervical biopsy |
| 67.12 | cpt dx | Other cervix biopsy |
| 67.19 | cpt dx | Other diagnostic procedures on cervix |
| 67.1 | cpt dx | Diagnostic procedure on cervix |
